# Determinants of Frugal Behavior: The Influences of Consciousness for Sustainable Consumption, Materialism, and the Consideration of Future Consequences

**DOI:** 10.3389/fpsyg.2020.567752

**Published:** 2020-11-23

**Authors:** Ernesto Suárez, Bernardo Hernández, Domingo Gil-Giménez, Víctor Corral-Verdugo

**Affiliations:** ^1^Departamento de Psicología Cognitiva, Social y Organizacional, Universidad de La Laguna, San Cristóbal de La Laguna, Spain; ^2^Departamento de Psicología y Ciencias de la Comunicación, Universidad de Sonora, Hermosillo, Mexico

**Keywords:** consciousness, frugality, future, materialism, sustainable consumption

## Abstract

The transition toward sustainability and the adjustment to climate change should involve the reduction of consumption behavior and the need to maintain social practices of frugality. This paper investigates the influences of consciousness for sustainable consumption (CSC), materialism, and the consideration of future consequences (CFC) on frugal behaviors. Four-hundred-and-forty-four individuals responded to an instrument investigating these variables. Results of a structural model revealed that materialism significantly and negatively influenced the three dimensions of CSC: economic, environmental, and social. The consideration of distant future consequences positively and significantly affected the economic dimension of CSC. Frugal behavior received significant and positive influences from the three CSC dimensions and from consideration of distant future consequences. The model explained 46% of variance in frugal behavior, revealing the importance of awareness of the consequences of resource consumption and the CFC has on promoting a moderate consumption of resources.

## Introduction

Mitigation and adaptation to climate change cannot be achieved without a reduction in the consumption of materials, products, and energy by the population of the world’s wealthiest countries. This, in turn, will not be accomplished without a considerable transformation in daily consumption patterns (De Young, 2014; Guillen-Royo and Wilhite, 2015). A special report by the Intergovernmental Panel on Climate Change (IPCC) on the impact of global warming, notes that human activities are the cause of the approximately 1.0°C increase in the total temperature of the planet, and estimates an increase of 1.5°C between 2030 and 2052 if global temperature continues to rise at the current rate (IPCC, 2018). Decreasing demand for energy and demand for goods linked to intensive land use and greenhouse gas pollution, would make it possible to limit warming as close as possible to 1.5°C, yet would still entail systemic changes on an unprecedented scale. Taken together, such actions illustrate an intentional, systematic, and significant reduction in consumption rates of goods and services. On a behavioral dimension, the transition toward sustainability and the adjustment to environmental conditions linked to climate change involve the reduction of consumption behavior and the need to maintain social practices of austerity and frugality. This study aims to advance the understanding of frugal behavior and to analyze how people’s awareness of the consequences of consumer behavior, materialism, and the consideration of the future consequences of their behavior affects the underlying conduct.

### Frugality as Lifestyle

Lastovicka et al. (1999, p. 96) defined frugality early on as a “lifestyle trait reflecting disciplined acquisition and resourcefulness in product and service use. Frugality is sacrifice in denying a series of short-term purchasing whims and industriousness by resourcefully using what is already owned or available for use; all of this is in service of achieving longer term goals.” Similarly, Michaelis et al. (2020) defined frugality as a consumer trait, which triggers a preference for conserving resources and applying economic rationality in their acquisition (i.e., assessing the opportunity cost of purchased goods and products).

As a lifestyle trait or orientation, frugality has been positively linked with voluntary simplicity and anticonsumption, and negatively with materialism. Iwata (1997, 2006) defined voluntary simplicity as a lifestyle of low consumption that includes low material dependency. However, although related, voluntary simplicity and frugality respond to different motivations (Pan et al., 2019). Thus, while voluntary simplicity is guided by a search for non-materialistic sources of life satisfaction and wellbeing, associated with values of self-determination, ecological awareness, and personal growth, frugality is explained by a set of specific consumer characteristics such as market mavenism and shopping antipathy (Bove et al., 2009).

Additionally, frugality is conceived as a lifestyle orientation opposed to materialism (Goldsmith and Flynn, 2015), to the extent that materialism involves the acquisition of goods as a means to achieve life goals, and “guides people’s choices and conduct in a variety of situations, including, but not limited to, consumption arenas” (Richins and Dawson, 1992, p. 307). This influences the quantity and quality of the goods that are purchased, suggesting that people who place a high value on material possessions and their acquisition will behave differently from those who place a lower value on them. Thus, while frugality is linked to constraints on consumption, materialism is linked to an increase in consumption. From a temporal perspective, frugality also implies a tendency to sacrifice short-term consumption to achieve longer-term social and personal goals, in contrast to materialism (Sung, 2017). There is, however, some evidence that questions this opposition between frugality and materialism. For example, Evers et al. (2018) found that both orientations positively influenced product end-consumption behaviors such as finding new uses for old products, and using a product differently than most other people.

It is possible to conceive frugality as a pattern of thoughts, feelings, and intentions that determine an individual’s preference for conserving resources and obtaining more from less, regardless of situational conditions, thus motivating frugal behavior (Goldsmith et al., 2014).

### Definition of Frugal Behavior

Frugal behavior implies a voluntary, deliberate, and proactive decision, not tied exclusively to circumstances associated with the economic structure and financial conditions (Muiños et al., 2015). It can be defined as a set of self-regulated consumer behaviors, related to both disciplined and restricted purchasing and the resourceful use of available products and services. These behaviors include routine actions such as seeking value and low prices, limiting consumption, using products as long as possible, taking care of and maintaining available goods, purchasing material goods considering future needs, repurposing, repairing, reusing, and recycling (Lastovicka et al., 1999; Goldsmith et al., 2014; Awais et al., 2020). Pepper et al. (2009) empirically operationalize the concept of frugal consumer behavior based on six items that highlight control during the purchase of goods. Yet this is strictly limited to “purchasing” behavior. Based on the approach of Lastovicka et al. (1999), Muiños et al. (2015) propose a 10-item scale that, in addition to the controlled purchase of goods, specifically operationalizes frugal behavior in terms of reducing the acquisition of goods along with an ingenious use of resources. However, few studies have been conducted addressing the behavioral analysis of frugality, not as a lifestyle or value orientation, but as a set of consumption reduction behaviors that, therefore, positively impact sustainability.

Based on the assumption that frugal behavior is anchored on the motivation to save while consuming goods and services, it is feasible to consider its positive association with environmentally sustainable actions that involve saving and conserving natural resources. From this perspective focused on consumption, frugal behavior and pro-environmental behavior maintain a close theoretical and empirical relation but also diverge on key aspects. Both types of behavior are influenced by similar cognitive and emotional processes (Hernández et al., 2012; Tapia-Fonllem et al., 2013) and by a non-materialistic orientation toward consumption (Pepper et al., 2009). However, it is possible to maintain a more lenient judgment on the environmental impact attributed to the quantity of products consumed when these have been identified as green/eco-friendly than when they have not been identified as such (e.g., Kim and Schuldt, 2018). As Akenji (2014) and Akenji and Bengtsson (2014) point out, it seems that “green consumption” could encourage over-consumption and make consumers feel less guilty, while at the same time driving them to take responsibility for maintaining economic growth, and conversely, driving the system toward sustainability (Akenji, 2014). In terms of consumer behavior, the relationship between frugal and environmental behaviors is, in this sense, neither direct nor simple.

For example, when studying the effect of future consequences, affinity toward diversity, altruism, environmental emotions, and pro-sustainability predisposition (personal willingness to ensure the long-term reciprocal sustainability of human-nature interactions), Corral-Verdugo et al. (2009) identify different factor loadings for pro-environmental behavior (0.81), altruistic behavior (0.62), and austere (frugal) behavior (0.52). While pro-environmental behavior is strongly explained by this group of factors, frugal behavior is explained to a far lesser extent.

Empirical research presents contradictory evidence regarding the role of environmental concern in the frugal reduction of consumption. Kropfeld et al. (2018) compared the influence of three anti-consumption lifestyles (voluntary simplicity, frugality, and tightwadism) and the environmental concern associated with consumption of 27 products and services with different levels of ecological impact. Their results showed that voluntary simplicity and tightwadism presented a greater relation with lower ecological impact than environmental concern, whereas frugality was not associated with reduced impact after demographic variables were controlled for. Furthermore, in a survey study conducted in 10 European countries with a sample of 1,000 people per country, Thøgersen (2018) found that attitudes toward energy saving mediated the relationship between environmental and frugal self-identities and energy-saving behaviors at home. However, the correlation between frugal and environmental identity is only moderate. In fact, in three of the 10 countries, while a direct effect of environmental self-identity on behavior is observed, no direct effect by frugal identity is identified after controlling for attitude.

When clarifying this contradictory evidence, it is necessary to take into account the level of specificity of the determinants of pro-environmental and frugal behavior. The concept of responsible consumption emphasizes consumption behaviors oriented by ethical, social, and/or ecological reasons or motives (Pepper et al., 2009). From this point of view, it is interesting to analyze the extent to which frugal behavior is determined, in particular, by the awareness of the consequences of consumption on ecological and social sustainability.

### Consciousness for Sustainable Consumption and the Consideration of Future Consequences as Determinants of Frugal Behavior

Balderjahn et al. (2013, p. 182) developed a comprehensive measurement of the consciousness for sustainable consumption (CSC). CSC is defined as “an intention to consume in a way that enhances the environmental, social, and economic aspects of quality of life.” In this case, consciousness was operationalized by weighting personal beliefs with the importance consumers attach to these three dimensions of sustainability. First, the environmental dimension implies that consumer consciousness is anchored on an environmentally friendly concern, specifically on five key environmental factors of consumption: (1) recycling, (2) packaging, (3) resources and energy, (4) local production, and (5) climate. Second, the social dimension assumes that a socially responsible consumer bases his or her purchase and usage of products on the desire to minimize or eliminate negative consequences on society, while being motivated to consume by the desire to do something positive for others. And third, the economic dimension could be summarized as the conscious effort to take care of personal economic well-being in the long-term. This dimension, in turn, is made up of three motivational dispositions that influence the decision of whether or not to buy: voluntary simplicity, debt-free consumption, and an interest in collaborative consumption.

Awareness of the environmental, social, and economic consequences of consumption act as a direct precedent for personal and societal responsible consumer behaviors (Buerke et al., 2017). Thus, CSC is distinctly related with different consumption and purchasing profiles, both sustainable and unsustainable (Peyer et al., 2017; Balderjahn et al., 2018). In any case, it is necessary to consider that the weight of each of the three dimensions on consumption decisions is also different (Hüttel et al., 2018).

The interest in establishing the relationship between time perspective, frugal, and pro-environmental behaviors is linked to the idea that environmental problems are the product of temporal conflicts between short-term and long-term benefits (Joireman et al., 2004). There is consistent empirical evidence of a moderate relation between future time perspective and pro-environmental behaviors (see, Milfont et al., 2012), and the influence of the consideration of future consequences (CFC). Less evidence is available on the link between CFC and frugal behavior and voluntary simplicity (e.g., Chang, 2018). “The CFC refers to the extent to which individuals consider the potential distant outcomes of their current behaviors and the extent to which they are influenced by these potential outcomes. It involves the intrapersonal struggle between present behavior with one set of immediate outcomes and one set of future outcomes” (Strathman et al., 1994, p. 743). Strathman et al. (1994) developed the CFC Scale, which includes elements that express a concern for more future consequences and others expressing a concern for more immediate consequences or a tendency to ignore future concerns. Pro-environmental behavior is moderated by this distinction between considerations of immediate and distant consequences.

For example, the preference for biofuel over gasoline was inversely related to the consideration of immediate consequences and positively related to the consideration of distant consequences (Khachatryan et al., 2013). Similarly, climate change acceptance and commitment to engage in mitigation actions was influenced by CFC-distant, yet, consideration of immediate consequences produced no effect (Corral-Verdugo et al., 2017). In contrast, Arnocky et al. (2014) found that lower levels of CFC-immediate predicted environmental concern and motivation for pro-environmental behavior, but that there were no significant effects by CFC-distant. On a theoretical level, Joireman and King (2016) argue that it is possible to identify two pathways of CFC’s influence on behavior. The authors distinguish between the influence of a model of awareness and one of concern. The awareness pathway suggests that CFC impacts behavior through awareness of the consequences of one’s own behavior. The concern pathway suggests that CFC moderates the impact of relevant consequences on behavior.

The importance of the relation between considering the future consequences of behavior and sustainable consumption lies in the fact that frugal behavior, as an element of sustainability, requires this type of consideration if it is to be truly maintained over time. To this end, this study analyzes the multidimensional model of CSC proposed by Balderjahn et al. (2013) and consumers’ awareness of the future and the environmental consequences of their conduct as determinants of frugal behavior.

Research in environmental psychology has thoroughly analyzed a wide range of pro-environmental behaviors. Among these, green or eco-responsible consumption has received special attention, yet frugal behavior has scarcely been addressed. As frugal behavior is increasingly considered a component of responsible consumption and sustainability, analyzing and explaining this type of behavior is a challenge for environmental psychology. It is possible to meet this challenge by building on previous constructs on pro-environmental behavior and responsible consumption, and by expanding our knowledge on these constructs, better understanding frugal behavior. With this double purpose, based on the research mentioned above, we developed a model that combines the main variables described in those studies with frugal behavior.

Following previous research on the influence of awareness of the environmental, social, and economic consequences of consumption on responsible consumer behaviors (Buerke et al., 2017; Balderjahn et al., 2018), the model used in this study establishes CSC, and its environmental, social, and economic dimensions, as a determinant of frugal behavior. Furthermore, following the approach of Hüttel et al. (2018), the three-dimensional characterization of CSC facilitates a hypothetical differentiation of the effect of each dimension in explaining frugal behavior.

In addition, based on research on time perspective and empirical evidence on the relationship between this dimension and pro-environmental behavior, the model establishes that CFC will influence frugal behavior directly and indirectly, through CSC. Specifically, the model seeks to assess the impact on frugal behavior of the awareness pathway for CFC defined by Joireman and King (2016). In both cases, however, differentiating a positive relation between distant future and frugal behavior and a negative relation between immediate future and frugal behavior.

Furthermore, the relation between CSC and CEC will be positive regarding distant future consequences and negative with immediate future consequences. Prior evidence of a significant effect on behavior from immediate and distant future considerations is contradictory (e.g., Arnocky et al., 2014; Corral-Verdugo et al., 2017). However, sustainable consumption implies that people take into account the relationship between short-term and long-term benefits of their consumption. It is, therefore, reasonable to hypothesize that CSC is linked in opposite ways to both dimensions of future considerations.

Lastly, following research on materialism and consumption behavior highlighting how materialistic people place greater importance on the acquisition of material goods as a means of achieving life goals and increasing subjective well-being (Richins and Dawson, 1992; Goldsmith and Flynn, 2015), materialism is linked to a present time perspective and is positively associated with excessive and compulsive consumption (Ku et al., 2018; Unger et al., 2018). In contrast, frugality and frugal behavior are strongly and positively associated with a future time perspective but are not related to a present time perspective (Dholakia et al., 2016). Therefore, it is feasible to consider a negative relation between materialism and time perspective. Taking this into account, the model used in this study establishes that materialism is negatively related to CFC and CSC.

Specifically, we establish the following hypotheses regarding the explanation of frugal behavior, and regarding the relationships between the factors previously mentioned, that determine frugal behavior:

- Frugal behavior is significantly, directly, and positively related to the three dimensions of consciousness for sustainable consumption, environmental, social and economic (H1).
- Consideration of distant future consequences is related to frugal behavior in a positive and significant way (H2).
- Consideration of immediate future consequences is negatively related to frugal behavior (H3).
- Materialism is negatively related to consciousness for sustainable consumption (H4), and consideration of future consequences, distant and immediate (H5 and H6).
- Consciousness for sustainable consumption, in its three factors, has a positive relation with consideration of distant future consequences (H7) and a negative relation with consideration of immediate future consequences (H8).

**Figure 1:**
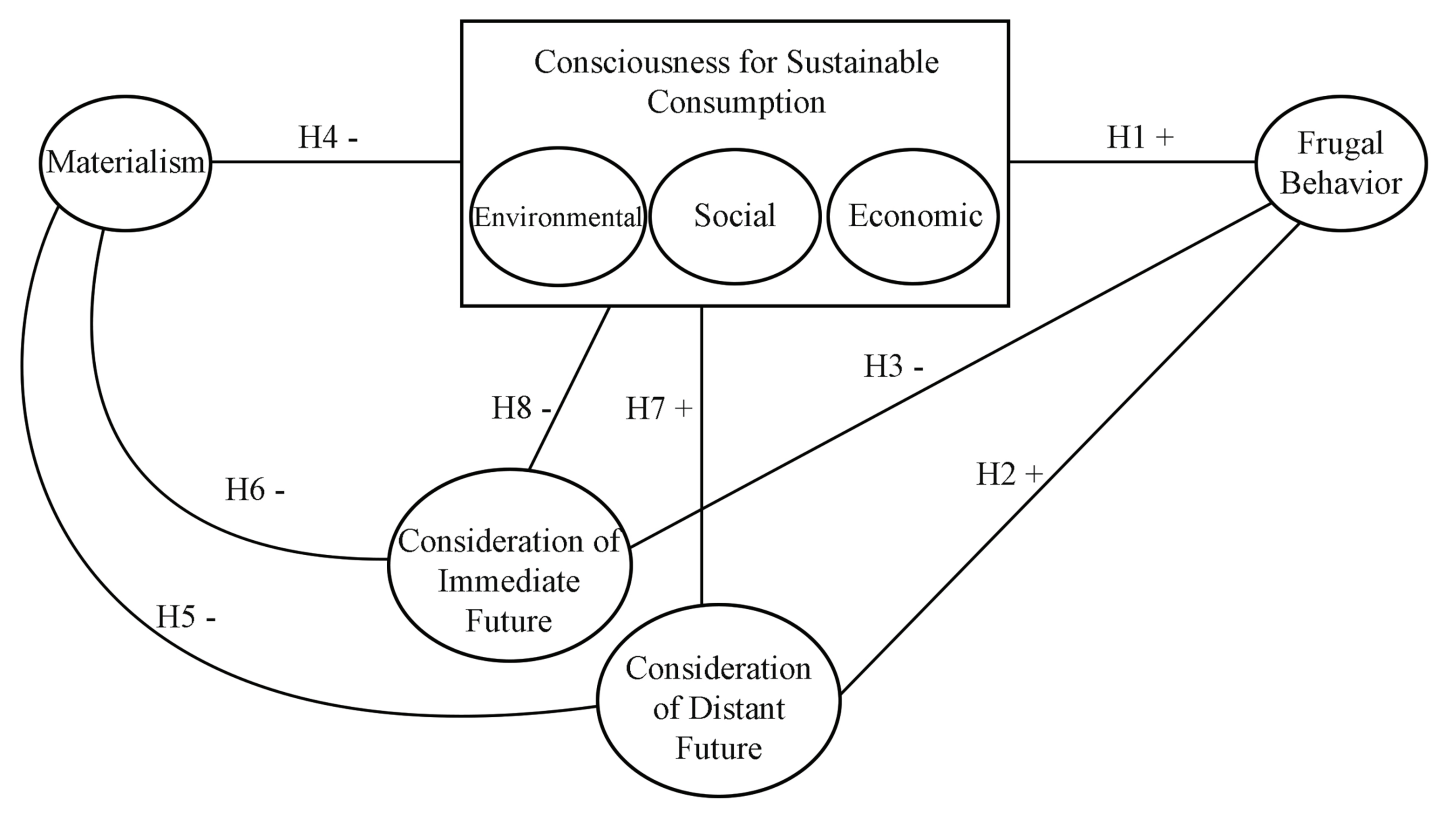
Summary of hypotheses.

Figure 1 summarizes the hypotheses tested in this study.

## Materials and Methods

### Participants

The total sample consisted of 444 people, of whom 282 were women and 162 were men, aged between 18 and 81 (*M* = 35.02 years, *SD* = 15.09), all were residents of Tenerife, Canary Islands, Spain. The composition of the sample followed data provided by the Canary Islands Institute of Statistics (ISTAC, 2019) regarding distribution by age group, educational level, monthly family income and employment status. Twenty-four and five tenths percentage stated that they had completed secondary education, 32% were studying at university, and 30.6% had already completed their studies. Regarding participants’ employment status, 48.9% were working, 9% were unemployed, 34.9% were studying, and 7.2% were retired. Twenty-one and six tenths percentage of the participants stated that they had a family income of less than 1,000€ per month, 30.4% between 1,001 and 1,500€, 21.6% between 1,501 and 2,000€, 9.2% between 2,001 and 2,500€, 6.8% between 2,501 and 3,000€, and finally, 10.1% stated that they had an income of more than 3,000€ per month.

Participants were first informed about the objectives of the study, the approximate duration of the participation, and the option of refusing to participate at the beginning of the questionnaire. Second, participants were informed that their personal data and information provided during the study would be stored securely, and that it would be processed with confidentiality, in accordance with data protection regulations. Finally, they were asked for their consent to participate in the survey and were informed that they could stop participating at any point if they chose to do so. The procedure followed complied with the principles of the Helsinki Declaration for research with human beings.

### Instruments

In this research, an instrument composed of different scales was used to measure frugal behavior, CFC, CSC, and materialistic values, all used a Likert-type response with scores from 1 to 7.

#### Frugal Behavior Scale (Muiños et al., 2015)

The Frugal Behavior scale is composed of 10 items and is an adaptation of the scale proposed by Lastovicka et al. (1999). It measures voluntary restriction of the acquisition of goods along with an ingenious use of the resources already available to the individual. Examples of the items in this scale are: “I look after my belongings to save in the long term,” “If I can re-use something that I already have, I do not buy anything new,” or “In order to save money, I am willing to wait before buying something I want.”

#### Consideration of Future Consequences Scale (Strathman et al., 1994)

The CFC scale is adapted to Spanish and validated by Echeverría et al. (2017) and Vásquez-Echeverría et al. (2018). It measures the extent to which people consider the consequences of their behavior in time as more distant vs. immediate. The scale consists of 14 items divided into two factors or subscales, consideration of immediate future consequences (CFCi), and consideration of distant future consequences (CFCf).

#### Consciousness for Sustainable Consumption Scale (Balderjahn et al., 2013)

The CSC scale measures the intention to consume in such a way that improves the environmental, social, and economic aspects of quality of life. The scale consists of 19 items, grouped into three dimensions: environmentally friendly consumption (ENV), socially responsible and fair consumption (SOC), and economically sustainable consumption (ECON). The latter is a second-order construct composed of three factors: voluntary simplicity (SIMP), debt-free consumption (NODEBT), and collaborative consumption (COLLAB). The 19 items repeat themselves, first to measure the beliefs associated with these factors and second to measure the importance assigned to them. The final score for each item results from multiplying the value obtained by measuring belief by the value obtained by measuring importance.

#### Materialism Scale (Richins, 2004)

The Materialism scale measures the importance given to the acquisition and possession of material goods as a means of achieving vital goals or objectives. The short version developed by Richins was used, composed of nine items, designed to assess materialism at a general level. An initial adaptation to Spanish of the Materialism scale was done, following guidelines by Muñiz et al. (2013), for its use in this study. The items were translated from English to Spanish and again from Spanish to English by two independent experts, to verify that both the meaning of the items and their intentionality are maintained in the Spanish version.

### Data Analysis

Data were treated using IBM SPSS Statistics for Windows, version 24.0 and EQS structural equation modeling software 6.1 for Windows. Scores were initially defined for each item in the CSC scale by calculating the product obtained from multiplying the values from the set of items assessing beliefs by the set of items that measure importance. The internal consistencies of all scales were then estimated, as well as the mean scores for each scale and the correlations between them.

Subsequently, a confirmatory factor analysis (CFA) of the CSC scale was conducted: five first-order factors (“environmental,” “social,” “simplicity,” “debt-free,” and “collaborative”) and one second-order factor (“economic”) were specified and tested. Three parcels were computed to act as indicators for every first-order factor. A parcel is the mean of two or more randomly chosen items (from the total set of items conforming the scale) of a construct (Little et al., 2002; Li et al., 2020). The “economic” second-order factor was specified as emerging from the interrelations between the “simplicity,” “debt-free,” and “collaborative” dimensions. Covariances between the three exogenous factors (“environmental,” “social,” and “economic”) of this model were calculated. Goodness-of-fit indicators were also obtained to determine the adequacy of this factorial representation.

Furthermore, a CFA of the Materialism scale was conducted. Given that Richins (2004) establishes the short version of the scale as a measure designed to assess materialism on a general level, a one-factor solution of the scale is tested.

Finally, a structural equation model was specified and tested. In this model, the six previously described factors were included along with four additional variables: (1) “Materialism,” an index computed from the average responses to the Materialism scale, (2) “Immediate Future,” computed as the mean of the Consideration of Immediate Future Consequences sub-scale, (3) “Distant Future,” the average of the Consideration of Distant Future Consequences sub-scale, and (4) “Frugal Behavior,” the mean of the Frugal Behavior Scale. The model specified direct influences from materialism, immediate and distant future on economic, social and environmental CSC; in addition, the two dimensions of future consequences and the three CSC factors were specified as directly affecting frugal behavior. The covariances between the three exogenous (materialism, distant future, and immediate future) factors were computed as well as the goodness-of-fit indicators for this model.

## Results

The Consciousness for Sustainable Consumption scale combines the degree to which respondents hold a belief with the significance or importance that belief has to the person. Given the structure of the scale, the means and standard deviations of each item were identified separately, depending on whether beliefs or importance were being measured in regard to the different dimensions of sustainable consumption. Table 1 presents the statistics for each item, as well as the result of multiplying belief and importance.

**Table 1:** Means and standard deviations of beliefs, importance, and the result of multiplying both set of items of the consciousness for sustainable consumption (CSC) scale.

Belief	*M*	*SD*	Importance	*M*	*SD*	Total	*M*	*SD*
ENV1	3.361	1.666	ENV1	5.003	1.699	ENV1	18.093	12.051
ENV2	4.281	1.840	ENV2	5.268	1.664	ENV2	23.947	13.808
ENV3	3.859	1.732	ENV3	5.172	1.637	ENV3	21.325	13.018
ENV4	4.072	1.725	ENV4	5.274	1.613	ENV4	22.733	12.943
SOC1	4.308	1.858	SOC1	5.653	1.607	SOC1	25.508	14.007
SOC2	4.695	2.062	SOC2	5.835	1.652	SOC2	28.890	15.707
SOC3	4.436	1.949	SOC3	5.726	1.636	SOC3	26.827	14.803
SOC4	4.576	1.985	SOC4	5.773	1.666	SOC4	27.850	15.180
SOC5	4.487	1.965	SOC5	5.713	1.607	SOC5	27.099	14.767
SIMP1	5.610	1.324	SIMP1	5.985	1.107	SIMP1	34.117	11.457
SIMP2	5.903	1.140	SIMP2	6.016	1.090	SIMP2	36.127	10.899
SIMP3	5.856	1.286	SIMP3	6.027	1.063	SIMP3	35.998	11.547
NODEBT1	6.034	1.423	NODEBT1	6.013	1.360	NODEBT1	37.190	13.228
NODEBT2	5.787	1.469	NODEBT2	6.055	1.294	NODEBT2	35.970	12.851
NODEBT3	5.429	1.510	NODEBT3	5.727	1.418	NODEBT3	32.233	13.268
NODEBT4	5.671	1.499	NODEBT4	5.884	1.394	NODEBT4	34.582	13.543
COLLAB1	5.999	1.585	COLLAB1	5.976	1.511	COLLAB1	37.160	14.028
COLLAB2	3.903	2.061	COLLAB2	4.511	2.095	COLLAB2	20.524	15.702
COLLAB3	4.759	1.877	COLLAB3	4.935	1.945	COLLAB3	25.795	15.812

ENV, environmental factor; SOC, social factor; SIMP, voluntary simplicity; NODEBT, debt-free consumption; COLLAB, collaborative consumption.

The internal consistency of the total scale and of the different subscales that make up CSC were then calculated. A Cronbach alpha of 0.871 was obtained for the total scale, while the following scores were obtained for corresponding factors that compose the scale: ENV with four items 0.935, SOC with five items 0.978, SIMP with three items, 0.842, NODEBT with four items 0.898, and COLLAB with three items 0.738. The ECON factor, composed of SIMP, NODEBT, and COLLAB, obtained an alpha of 0.872. After the alpha scores were obtained, the mean scores for each of the variables analyzed were computed. These results are shown in Table 2.

**Table 2:** Descriptive and alpha values of the used scales.

Scales	Alpha	*M*	*SD*
Fr	0.876	5.536	0.960
CFCi	0.777	2.863	0.993
CFCf	0.694	5.140	0.860
CSC	0.871	5.252	0.784
ENV	0.935	4.536	1.350
SOC	0.978	5.120	1.475
ECON	0.872	5.604	0.873
SIMP	0.842	5.899	0.866
NODEBT	0.898	5.825	1.087
COLLAB	0.738	5.014	1.357
MAT	0.856	3.036	1.136

Scores are presented on the same response scale that participants used when responding (1–7). Fr, frugality; CFCi, consideration of immediate future consequences; CFCf, consideration of distant future consequences; CSC, consciousness for sustainable consumption; ENV, environmental factor; SOC, social factor; ECON, economic factor; SIMP, voluntary simplicity; NODEBT, debt-free consumption; COLLAB, collaborative consumption; MAT, materialism.

Regarding the reliability of the rest of the scales used (Table 2), the subscale of Consideration of Immediate Future Consequences (CFCi), composed of seven items obtained a Cronbach alpha of 0.777. The Consideration of distant Future Consequences (CFCf) subscale produced a Cronbach alpha of 0.694, with seven items. The Frugal Behavior scale, composed of 10 items, obtained a Cronbach alpha of 0.876, and the Materialism scale, composed of nine items, produced a Cronbach alpha of 0.856. Pearson’s correlations were also estimated between all variables; the results are shown in Table 3.

**Table 3:** Correlation matrix between the scales.

Scales	1	2	3	4	5	6	7	8	9	10
Fr										
CFCi	−0.219^**^									
CFCf	0.436^**^	−0.270^**^								
CSC	0.325^**^	−0.172^**^	0.143^**^							
ENV	0.102^*^	−0.063	−0.031	0.694^**^						
SOC	0.004	−0.049	−0.036	0.725^**^	0.695^**^					
ECON	0.455^**^	−0.199^**^	0.269^**^	0.637^**^	0.020	−0.012				
SIMP	0.463^**^	−0.201^**^	0.267^**^	0.498^**^	0.137^**^	0.027	0.689^**^			
NODEBT	0.385^**^	−0.215^**^	0.210^**^	0.626^**^	0.074	0.081	0.881^**^	0.442^**^		
COLLAB	0.264^**^	−0.059	0.184^**^	0.384^**^	−0.152^**^	−0.152^**^	0.802^**^	0.341^**^	0.558^**^	
MAT	−0.246^**^	0.314^**^	0.009	−0.300^**^	−0.227^**^	−0.132^**^	−0.249^**^	−0.344^**^	−0.253^**^	−0.019

Fr, frugality; CFCi, consideration of immediate future consequences; CFCf, consideration of distant future consequences; CSC, consciousness for sustainable consumption; ENV, environmental factor; SOC, social factor; ECON, economic factor; SIMP, voluntary simplicity; NODEBT, debt-free consumption; COLLAB, collaborative consumption; MAT, materialism.

** <0.01;

* <0.05.

**Figure 2:**
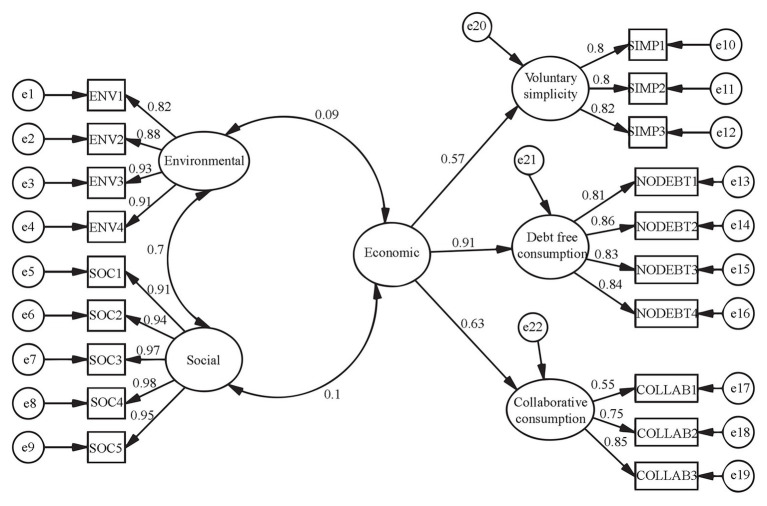
Confirmatory factor analysis (CFA) for the CSC scale. *N* = 496, *χ*^2^ = 550.315 (146 *df*), *p* = 0.000, *CFI* = 0.95, *RMSEA* = 0.75.

Figure 2 presents the results of the CFA of the CSC scale. The five first-order factors (“environmental,” “social,” “simplicity,” “debt-free,” and “collaborative”) and the “Economic” second-order factor coherently emerged from the interrelations between their corresponding indicators. Salient (from 0.55 to 0.98) and significant (*p* < 0.05) lambdas between every factor and their indicators evidenced convergent construct validity for the instruments used. Voluntary simplicity, debt-free and collaborative dimensions of the CSC produced the “Economic” latent variable, as revealed by their factorial loadings on this second-order factor. A high covariance (0.70) resulted between the social and environmental CSC dimensions; however, the relations between these factors and the economic dimension were rather small (0.10, and 0.09, respectively), yet significant (*p* < 0.05). The CFI practical-goodness-of-fit indicator (0.95) and the root means square error of approximation (RMSEA; 0.07) revealed that this CFA is supported by the data.

Additionally, a CFA of the Materialism scale was conducted. The one-factor model proposed is supported by the data, with acceptable goodness-of-fit indicators, with CFI (0.97) and RMSEA (0.06).

**Figure 3:**
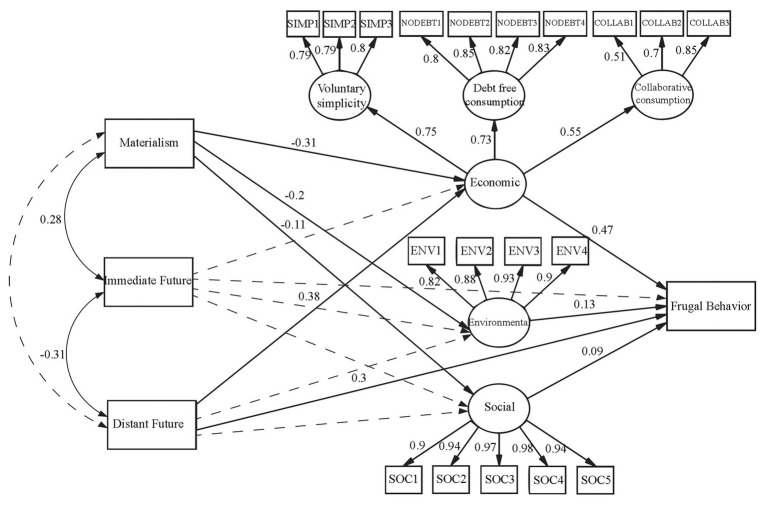
Model of frugal behavior explained by CSC, materialism, and consideration of future consequences (CFC). *N* = 444, *χ*^2^ = 1030.75 (213 *df*), *p* < 0.0001, *BNNFI* = 0.89, *CFI* = 0.90, *RMSEA* = 0.09, *R*^2^ = 0.46.

Finally, Figure 3 shows the results of the structural model. As anticipated, materialism significantly and negatively influenced the economic (structural coefficient = −0.31), environmental (−0.20), and social (−0.11) dimensions of CSC (Hypotheses 4 is supported). Materialism was also significantly and positively related to consideration of immediate future consequences (0.28; Hypothesis 6 is rejected) but was not related to consideration of distant future consequences (Hypothesis 5 is rejected). The consideration of distant future consequences positively and significantly affected CSC, but only its economic dimension (structural coefficient = 0.38), however, neither the social or environmental dimensions were significantly influenced (Hypothesis 7 is partially supported). The consideration of immediate future consequences did not impact any of the CSC factors (Hypothesis 8 is rejected). Frugal behavior, in turn, received significant and positive influences from the three CSC dimensions: the economic factor produced a 0.47 structural coefficient on that dependent variable, while the environmental (0.13) and social (0.09) dimensions marginally impacted frugal behavior (Hypothesis 1 is supported). Distant future contributed with a 0.30 structural coefficient (Hypothesis 2 is supported), but immediate future did not significantly impact frugal behavior (Hypothesis 3 is rejected). The model’s *R*^2^ was 0.46, *χ*^2^ = 1030.75 (213 *df*), *p* < 0.0001, and BNNFI = 0.89. The CFI practical-goodness-of-fit indicator (0.90) reveals an adequate fit for this model, and RMSEA was 0.09, close to the acceptable limit of 0.08.

## Discussion

The central aim of this study was to analyze the extent to which frugal behavior is influenced by people’s awareness of the consequences associated with consumption and the role played by the CFC and materialistic values. Our study confirms the multi-faceted definition of CSC proposed by Balderjahn et al. (2013), as the intention to consume in an environmentally friendly, socially-just, and economically sustainable way. As was hypothesized, the three dimensions of CSC were also confirmed as having a significant influence on frugal behavior.

In line with the model proposed by Balderjahn et al., we tested the factor structure of the CSC scale, presuming that five first-order factors (environmental, social, voluntary simplicity, debt-free, and collaborative consumption) would result from its item-interrelations. Such presumption was confirmed. In addition, the assumption that a higher-order “Economic” dimension is subjacent to voluntary simplicity, debt-free, and collaborative forms of consumption was confirmed, also revealing that the environmental and social aspects of CSC were relatively independent from the economic dimension. The covariance between the social and the environmental dimensions was moderately high (=0.70), suggesting that the pro-social and pro-environmental motives go together in individuals that are prone to consume in a sustainable way. However, those motives are not well connected to the economic dimension: the covariances between the social and environmental dimensions of CSC and the economic dimension were markedly small (0.10 and 0.09), implying a clear distinction between the economic and the socio/environmental intentions to consume in a sustainable way.

Our results differ partially from those obtained by Balderjahn et al. (2013). In their original paper, the authors reported that the economic dimension was significantly related to the environmental dimension and partially but not significantly to the social dimension. The CSC scale seems to be capable of capturing the three dimensions of consciousness traditionally associated with the concept of sustainability. However, our work also points to a relevant empirical gap in the eco-social and economic motivational basis of sustainable consumption. This result calls into question the idea of a single, perfectly integrated framework of beliefs about sustainable consumption. From this point of view, within the context of personal beliefs, it is feasible that the consideration of environmental, social, and economic consequences associated with consumption would not be activated either simultaneously or in a coherent manner.

This approach is consistent with the results of Balderjahn et al. (2018). In said paper, based on three German representative datasets, six consumer typology groups were identified, each of them oriented by a unique pattern of sustainable consciousness: (1) financially careless consumers, (2) non-simplifier consumers, (3) financially careful simplifiers, (4) socially conscious financial simplifiers, (5) sustainable, non-collaborative consumers, and (6) sustainable consumers. Each of these different consumer groups had a distinct sustainability consciousness. In fact, in each consumer segment, people’s multi-faceted sustainability concern also differed in its impact on purchases across different product categories. In other words, the environmental, social, and economic consequences of consumption are combined in different ways and also have a differential influence on the type and quantity of products purchased. In addition, it is also possible to identify contradictory interrelations such as consumers’ consciousness for collaborative behavior having a positive but weak contribution to non-sustainable economic behaviors like impulsive buying and high spending and indebtedness (Seegebarth et al., 2016).

A way of clarifying the role of awareness of the consequences of specific consumption practices on sustainability can be found in the analysis of CSC as an element or dimension of lifestyle. This is in line with Kropfeld et al. (2018), who point out that the adoption of anti-consumption lifestyles is not associated with a lower generalized consumption of goods and services but only with the reduction in consumption of certain and specific types of goods and services.

The results of the structural model revealed that, as stated in Hypothesis 4, materialistic beliefs negatively impacted the environmental, social, and economic dimensions of CSC, meaning that higher acceptance of materialism may result in a lower intention to consume sustainably, regardless of the (economic, environmental, or social) motives expressed by respondents. This is consistent with previous evidence that materialism is negatively associated with both pro-environmental attitudes and behaviors (e.g., Hurst et al., 2013). However, consideration of distant future consequences only influenced the economic facet of CSC, implying that, at least in our sample, people are concerned about the distant economic consequences of their current consumption but do not anticipate a significant impact of that consumption on environmental and social issues. Although it had been stated that consideration of distant future consequences would be linked to the three dimensions of CSC in a positive way (Hypothesis 7), the results obtained only show a significant and positive relationship with the economic dimension. A possible explanation of this result could be linked to the conceptualization of the CSC construct. The definition of the economic dimension of CSC, explicitly includes the idea of consuming in a manner that does not harm the long-term or future economic well-being of the individual. However, the environmental and social dimensions of CSC do not express an explicit focus on time perspective. In addition, contrary to Hypothesis 8, consideration of immediate future consequences had no effect on any of the three dimensions of CSC, implying that individuals who are more focused on the immediate future, disregard all aspects of CSC. It would, therefore, be of great interest to analyze the links between materialism and its dimensions (e.g., Nepomuceno and Laroche, 2015), and future perspectives, both immediate and distant. It had been hypothesized for this study that materialism would have a negative relation with both considerations of immediate and distant future consequences (Hypotheses 5 and 6). On the contrary, the results obtained showed a significant and positive relationship between materialism and immediate future consequences and no relation with distant future consequences. The positive relationship between materialism and immediate future could be partially explained by immediate future consequences being interpreted as too close to the present. Other studies have identified a link between materialism and present time perspective (e.g., Unger et al., 2018). In any case, the combination of a materialistic vision and the failure to anticipate the future consequences of consumer behavior may be one of the main barriers to socially promoting carbon footprint reduction.

Frugal behavior was significantly and positively affected by the economic, environmental, and social dimensions of CSC, confirming our Hypothesis 1, with the economic factor being the strongest predictor (structural coefficient = 0.47) of frugal behavior, in contrast with the environmental (0.13) and social (0.09) dimensions. Consideration of distant future consequences also influenced frugal behavior in a positive way, as stated in Hypothesis 2 (structural coefficient = 0.30). However, contrary to what was expected (Hypothesis 3), considerations of immediate future consequences was not linked to frugal behavior. Thus, frugality in consumption can be conceived, in line with what was proposed by Strathman et al. (1994), as a form of self-control, as it is associated with the consideration of the future consequences of one’s own behavior. Our results support the distinction between the immediate and distant factors of CFC (e.g., Joireman et al., 2008; McKay et al., 2016). In this sense, frugal behavior would be positively associated with the consideration of options maximizing long-term consequences and delaying rewards, and would be contrary to the tendency to discount the value of future outcomes associated with CFC-Immediate.

The fact that the economic dimension of CSC demonstrates a greater explanatory capacity over frugal behavior than its social and environmental dimensions, requires further exploration. It is necessary to bear in mind that certain economic factors have previously been identified as motivators of frugal behavior, such as market mavenism and shopping antipathy (Bove et al., 2009), interest in saving (Shoham et al., 2017) or brand engagement in self-concept (Goldsmith et al., 2014). However, if this result is replicated, it would indicate that people pay more attention to economic motives than to ecological or social consideration when deciding to engage in frugal ways of living. In any case, the four factors that produce significant effects on the model accounted for 46% of the variance in frugal behavior, a considerable amount of explained variance for a factor that deserves special attention in the study of sustainable behavior.

Although the different statistics used in this study support the reliability and validity of the instruments used and the adjustment of the proposed model, a limitation related to the RMSEA value must be noted. Specifically, the value obtained for this parameter was higher than desirable, suggesting a high amount of unexplained variance. However, taking into account that the RMSEA is particularly close to the recommended limit of 0.08 (Hu and Bentler, 1999) and that this parameter is highly dependent on the complexity of the model and the degrees of freedom (Muthén, 2001; Jackson, 2003; Kenny et al., 2015), it would be inappropriate to reject the model, given the weight of the rest of the statistics presented.

To summarize, our study makes at least three contributions to the literature. First, results showed that frugal behavior relies on the level of awareness people have regarding the multiple effects of consumption. Therefore, consciousness of the social, environmental, and economic consequences of consumption have been confirmed to influence frugal behavior, specifically consciousness of the economic consequences is the dimension that presents the greatest impact on frugal behavior. Second, the specific effect of the economic dimension on frugality can be understood as being related to the notion of personal consumption as a viewpoint that prioritizes economic independence. In this sense, it is possible to define the economic consideration of sustainable and frugal consumption from the concepts of voluntary simplicity, collaborative, and debt-free consumption, as well as having a negative relationship with materialism. Third, the importance of the temporal dimension in promoting and maintaining frugal behavior has been emphasized in this study. Specially, the consideration of distant future consequences has been identified as a fundamental component of the relationship between the temporal dimension and frugality.

This leads us to conclude that frugal behavior and pro-environmental behavior are handled differently. When considering intervention to promote frugal behavior, it becomes necessary to consider the influence of other factors such as materialism, CFC, or underlying economic factors, in addition to the consciousness of a social and environmentally sustainable consumption. Our research provides empirical evidence on the consistency and validity of the CSC scale as a novel instrument for measuring the intention to consume in a sustainable manner.

## Data Availability Statement

The raw data supporting the conclusions of this article will be made available by the authors, without undue reservation.

## Ethics Statement

Ethical review and approval was not required for the study on human participants in accordance with the local legislation and institutional requirements. The patients/participants provided their written informed consent to participate in this study.

## Author Contributions

ES, BH, DG-G, and VC-V contributed equally across all areas of the study, drafting the Introduction, Materials and Methods, Results, and Discussion sections, as well as conducting the data analysis. All authors approved the final version of the manuscript for submission.

### Conflict of Interest

The authors declare that the research was conducted in the absence of any commercial or financial relationships that could be construed as a potential conflict of interest.
